# Mechanical Activation of Graphite for Na‐Ion Battery Anodes: Unexpected Reversible Reaction on Solid Electrolyte Interphase via X‐Ray Analysis

**DOI:** 10.1002/advs.202401022

**Published:** 2024-04-26

**Authors:** Su Chan Lee, Young Hwan Kim, Jae‐Ho Park, Dieky Susanto, Ji‐Young Kim, Jonghyun Han, Seong Chan Jun, Kyung Yoon Chung

**Affiliations:** ^1^ Energy Storage Research Center Korea Institute of Science and Technology (KIST) Hwarang‐ro 14‐gil 5, Seongbuk‐gu Seoul 02792 South Korea; ^2^ Nano‐Electro Mechanical Device Laboratory School of Mechanical Engineering Yonsei University 50 Yonsei‐ro, Seodaemun‐gu Seoul 03722 South Korea; ^3^ Advanced Analysis Center Korea Institute of Science and Technology (KIST) Hwarang‐ro 14‐gil 5, Seongbuk‐gu Seoul 02792 South Korea; ^4^ Division of Energy & Environmental Technology, KIST School Korea University of Science and Technology Seoul 02792 South Korea

**Keywords:** anode, graphite, mechanical activation, Na‐ion battery, post‐mortem analysis, solid electrolyte interface, synchrotron X‐ray analysis

## Abstract

Although sodium‐ion batteries (SIBs) offer promising low‐cost alternatives to lithium‐ion batteries (LIBs), several challenges need to be overcome for their widespread adoption. A primary concern is the optimization of carbon anodes. Graphite, vital to the commercial viability of LIBs, has a limited capacity for sodium ions. Numerous alternatives to graphite are explored, particularly focusing on disordered carbons, including hard carbon. However, compared with graphite, most of these materials underperform in LIBs. Furthermore, the reaction mechanism between carbon and sodium ions remains ambiguous owing to the structural diversity of disordered carbon. A straightforward mechanical approach is introduced to enhance the sodium ion storage capacity of graphite, supported by comprehensive analytical techniques. Mechanically activated graphite delivers a notable reversible capacity of 290.5 mAh·g^−1^ at a current density of 10 mA·g^−1^. Moreover, it maintains a capacity of 157.7 mAh·g^−1^ even at a current density of 1 A·g^−1^, benefiting from the defect‐rich structure achieved by mechanical activation. Soft X‐ray analysis revealed that this defect‐rich carbon employs a sodium‐ion storage mechanism distinct from that of hard carbon. This leads to an unexpected reversible reaction on the solid electrolyte surface. These insights pave the way for innovative design approaches for carbon electrodes in SIB anodes.

## Graphical abstract

Graphite, vital to the commercial viability of lithium‐ion batteries (LIBs), has a limited capacity for sodium ions. Numerous alternatives to graphite are explored, particularly focusing on disordered carbons, including hard carbon. However, compared with graphite, most of these materials underperform in LIBs. A straightforward mechanical approach is introduced to enhance the sodium ion storage capacity of graphite, supported by comprehensive analytical techniques.

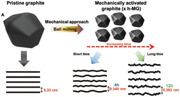

## 1 Introduction

Rechargeable batteries are indispensable in modern society, powering diverse applications on demand. Given the dwindling reserves and escalating cost of lithium, seeking alternatives has become imperative.^[^ ^1^ ^]^ Sodium‐ion batteries (SIBs) emerge as a leading contender to partially substitute prevalent commercial lithium‐ion batteries (LIBs) and broaden battery applications, given sodium's plentiful reserves, affordability, and performance similarities to LIBs.^[^ ^2^ ^]^ Identifying anode materials with high capacity and consistent cyclability is crucial for their practical implementation.

Graphite is frequently highlighted as a potential anode material for SIBs, drawing from its commercial success in high‐performance LIBs due to its energy density and low cost.^[^ ^3^ ^]^ However, natural graphite demonstrates subpar performance (35 mAh·g^−1^) with a limited reversible capacity for sodium ion storage.^[^ ^4^ ^]^ This issue stems from the larger ionic radius of sodium ions (0.102 nm) compared to lithium ions (0.076 nm), rendering the interlayer spacing of graphite (0.335 nm) insufficient for sodium ion storage.^[^ ^5^ ^]^ Computational studies further indicate that sodium ion insertion into graphite is not thermodynamically favored.^[^ ^6^ ^]^

Several approaches have been reported to integrate sodium ions in graphite: 1) transitioning from carbonate‐based solvents to ether‐based electrolytes in SIBs, and 2) enhancing graphite‐based anode materials through heteroatom doping.^[^ ^7^ ^]^ Although several studies on graphite SIB anodes have yielded significant findings, these anodes possess intrinsic limitations, including limited capacity, rate capability, and compatibility with diverse electrolytes.

To address these issues, remarkable endeavors have been directed toward designing and fabricating expanded graphite (EG), enabling sodium ion intercalation within the graphite layer.^[^ ^5^, ^8^ ^]^ Prior research aimed at augmenting the interlayer spacing of graphite has achieved promising results to an extent. For instance, Wen et al. reported that chemically EG prepared by heat‐treating graphene oxide possesses an expanded interlayer distance of 4.3 Å. This modified graphite displayed a capacity of 184 mAh·g^−1^ at 100 mA·g^−1^ when used with a polycarbonate‐based electrolyte.^[^ ^9^ ^]^ Past studies indicate that expanding the interlayer distance of graphite leads to improved reversible capacity, rate capability, and cycle stability. However, these enhancements required complex multi‐step processes with low yields. Therefore, crafting a straightforward and scalable method for producing EG becomes vital for practical applications.

From an electrochemical standpoint, while there have recently been numerous advancements in refining the performance of graphite anodes, the underlying reaction mechanism within these anodes remains unclear.^[^ ^10^ ^]^ The unidentified nature of the sodium‐ion storage mechanism in graphite has stymied further advancements in graphite‐based anode development. Exploring the reaction dynamics of Na^+^ ions in graphite chiefly concerns the electrochemical attribute alterations stemming from structural modifications during recurring intercalation/deintercalation.

Conversely, the reaction mechanism of sodium ions in hard‐carbon anodes has been documented. The voltage profiles observed in the charge/discharge curves of hard carbon anodes comprise two distinct regions: (1) a sloping voltage range (1.0–0.1 V vs Na/Na^+^) and (2) a plateau segment (≈ 0.1–0 V vs Na/Na^+^). Numerous studies posit that the reaction mechanism depicted in the voltage profile during charge/discharge is contingent upon the evolving structural properties of hard carbon. Stevens et al. introduced the “house of cards” model, proposing that during the sloping voltage phase, sodium ions insert into the graphite layer, and in the plateau voltage region, nanopores fill with metallic sodium.^[^ ^11^ ^]^ However, Liu et al. suggested a different “adsorption‐insertion” mechanism. They proposed that the sloping voltage phase corresponds to Na^+^ ion absorption at active sites, such as defects on the hard carbon surface, while the plateau voltage phase predominantly relates to sodium ion intercalation into the graphite layers.^[^ ^12^ ^]^ In addition, Lu et al. reported that the ball milling of hard carbon increased the amount of defects and reduced the crystal size. These results, which demonstrated reduced capacitance and initial efficiency at low potentials, also supported the “adsorption‐insertion” mechanism.^[^ ^13^ ^]^

However, hard carbon presents challenges, including diminished rate capability due to its limited conductivity and structural stability. Therefore, there is an urgent need to elucidate the reaction mechanism of sodium ions in graphite during charge/discharge. This is the inaugural comprehensive study examining the sodium‐ion reaction mechanism in graphite across the sloping and plateau voltage regions.

Recently, the ball milling of graphite has increased its viability as a host for sodium ions by controlling its particle size, surface area, and interlayer.^[^ ^14^ ^]^ Using the ball milling technique, we introduced a unique mechanical approach to prepare activated graphite. The physicochemical properties of the graphite were modulated based on the ball‐milling duration. We examined the sodium ion storage behavior of this mechanically activated graphite, focusing on alterations in its structural properties. The activated graphite showcased a capacity of 245 mAh·g^−1^ at a current density of 100 mA·g^−1^. Moreover, it maintained a capacity of 130 mAh·g^−1^ at an elevated current density of 1 A·g^−1^ and demonstrated outstanding cycling stability, sustaining 130 mAh·g^−1^ over 3000 cycles at a current density of 1 A·g^−1^. We employed near‐edge X‐ray absorption fine structure (NEXAFS) spectroscopy using synchrotron X‐rays to precisely discern the reaction mechanism within the sloping voltage region. These findings highlight an unforeseen reversible reaction at the solid electrolyte interphase (SEI) surface. Ex situ and in situ analyses revealed varied surface‐bulk reactions during ball milling. This newly posited reaction mechanism offers valuable perspectives for crafting and advancing high‐performance graphite anodes for SIBs.

## 2 Results and Discussion

### 2.1 Changes in the Structure and the Chemical Properties of Graphite

**Figure 1:**
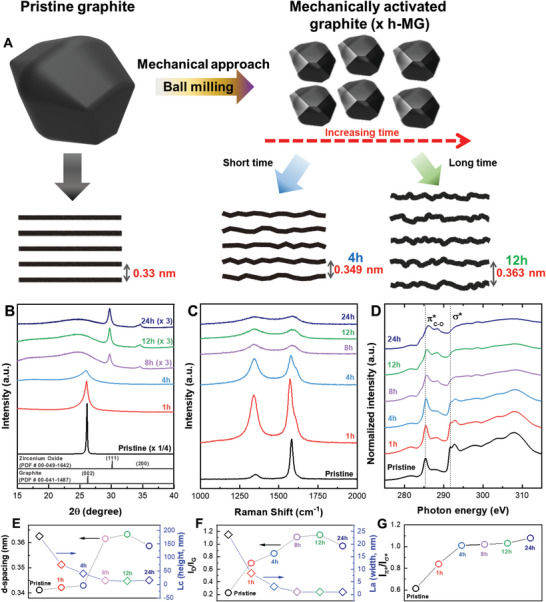
A) Schematic illustrations of pristine and ball‐milled graphite, along with interlayer structure representations before and after ball milling. B) X‐ray diffraction (XRD) patterns, C) Raman spectra, and D) near‐edge X‐ray absorption fine structure (NEXAFS) measurements for pristine graphite and ball‐milled graphite at different milling times. E) d‐spacing variation, F) crystallite size along the c‐axis (*L*_*c*_) calculated using the Scherrer equation from XRD, G) I_D_/I_G_ ratio in Raman spectra, and crystallite size along the a‐axis (*L*_*a*_) over milling time determined from XRD.

Ball milling influences the structural and physical attributes of graphite, including particle size and interlayer distance, alongside its chemical properties. Before delving into the impact of these modifications on the sodium‐ion storage capacity, we characterized the shifts in physicochemical properties corresponding to different ball‐milling durations. The samples are labeled as x h‐MG, where “x” represents the ball‐milling duration. For instance, “12 h‐MG” signifies graphite milled for 12 h (**Figure** **1**A). Crystal structure variations with ball milling time were assessed using powder X‐ray diffraction (XRD) (Figure 1B). Pristine graphite exhibited a pronounced diffraction peak at ≈26.12°, correlating to the (002) plane of graphite (PDF card No. 00‐049‐1642).^[^ ^15^ ^]^ With extended ball milling time, the intensity of this diffraction peak decreased. The diminished intensity and broadening of the (002) peak post‐milling signified a reduction in the crystallinity and particle grain size of the graphite. The diffraction peaks in the 8 h‐MG sample were notably fainter than those of pristine graphite. This suggests the emergence of a disordered carbon framework in the graphite, lacking a long‐range order.

The dotted lines in Figure 1B highlight the normalized intensities of the XRD peaks, illustrating variations in peak contours. Importantly, the (002) peak intensities decreased and subsequently flattened with increasing ball‐milling times; this is attributable to the increased defect density and decreased crystallinity because a high impact and shear force can deteriorate the C═C/C─C bonds in graphite. After 12 h of milling, a noticeable shift in the (002) peak toward a lower angle (26.4°) is observed. This shift reflects the swift amorphization of the structure and an increase in the interlayer spacing of the graphite layers. A distinct peak at 30.12°, observed post 8 h of milling, was ascribed to an unintentional ZrO_2_ impurity, likely sourced from the zirconia crucible and balls.^[^ ^16^ ^]^ The XRD data determined the crystallite size along the c‐axis of graphite, *L*_*c*_, and the interlayer spacing to elucidate the variations in particle size and crystalline structure. *L_c_* was computed using the Scherrer equation given by (Figure 1E):^[^ ^17^ ^]^

(1) $$\;L_{c}=\;0.9\frac{\lambda }{\beta \cos\theta }$$

where λ represents the X‐ray wavelength, θ is the Bragg angle, and β denotes the full width at half intensity of the diffraction peaks. Initially, pristine graphite exhibited an *L_c_* of 178.8 nm. However, after 4 h of milling, the *L_c_* of 4 h‐MG plummeted to 40.28 nm. Beyond 8 h of milling, the *L_c_* value was stabilized and remained constant. The *L_c_* values for 8 h‐MG, 12 h‐MG, and 24 h‐MG were calculated at 14.58, 13.09, and 15.57 nm, respectively. Approximately 80% of the total *L_c_* reduction occurred within the first 4 h of milling, with 20% alteration observed thereafter. The high‐energy ball milling can easily disrupt the innate order of the (002) basal planes owing to the weak bonds between these planes, reducing even large initial crystals to significantly reduced sizes post‐milling.

The interlayer distance of the (002) planes represents the spacing between the graphite layers and was determined from the position of the (002) diffraction peak using Bragg's equation:^[^ ^18^ ^]^

(2) 2dsinθ=nλ

In this equation, “d” represents the average interlayer spacing, “θ” represents the Bragg angle, and “λ” represents the X‐ray wavelength. Up to 4 h of milling, the d‐spacing remained relatively constant, with the d‐spacings of pristine graphite at 0.341 nm and 4 h‐MG at 0.343 nm. However, a sharp increase in d‐spacing becomes evident after 8 h of milling. The d‐spacings for 8 and 12 h‐MG were measured to be 0.361 and 0.363 nm, respectively. This expansion in interlayer distance was attributed to the insertion of interstitial carbon atoms between the aromatic planes of graphite due to mechanical milling. The ball milling equipment utilized in this study seemed to have reached its mechanical limit after 12 h, resulting in no additional grinding effect. For instance, the d‐spacing of 24 h‐MG, at 0.359 nm, was slightly lower than that of the 12 h‐MG. This could be due to the high‐temperature conditions within the ceramic vessels from frictional heat, potentially causing structural recovery at the atomic level of the milled graphite. The particle size rapidly decreased after 1 h of ball milling, while the inner crystal structure, as indicated by the d‐spacing, changed after 4 h.

Raman spectroscopy was conducted to investigate the degree of disorder and amorphization of the milled graphite, as shown in Figure 1C. Pristine graphite exhibits a prominent G peak (≈1580 cm^−1^) corresponding to sp^2^ hybridized carbon‐carbon bonds from the first‐order scattering of the E_2g_ phonon.^[^ ^19^ ^]^ However, the weak D peak (≈1350 cm^−1^) was associated with lattice distortions and defects within graphite.^[^ ^20^ ^]^ The intensities of the G and D peaks for pristine graphite indicate a well‐ordered structure with a defined crystal size. The D peak for the milled graphite was significantly larger than that for the pristine graphite, pointing to crystal size reduction and defect formation within the hexagonal graphite structure. The Raman spectra of graphite milled for 8 h are markedly different from those of pristine graphite. The intensity of the D peak surpasses that of the G peak, and both peaks broaden with a significant intensity decrease. The inverted intensity ratio between the D and G peaks was attributed to the considerable increase in the disorder of graphite caused by ball milling. This milling introduced lattice defects and exposed edges that inhibited lattice vibrations. The growing disorder degree was quantified using the I_D_/I_G_ ratio. The I_D_/I_G_ value escalated from 0.23 (pristine graphite) to its highest at 1.15 (12 h‐MG), subsequently decreasing to 0.97 after 24 h of milling. The I_D_/I_G_ ratio rise is attributed to the formation of structural defects and lattice disorder during milling; thus, 12 h‐MG could possess the highest interlayer distance and lowest crystalline size.

Furthermore, NEXAFS spectroscopy was utilized to examine alterations in the bonding structure of the milled graphite. Typically, graphite consists of stacked parallel carbon layers with different chemical bonds in the in‐plane and in‐plane directions.^[^ ^21^ ^]^ Each carbon atom forms a strong covalent sigma (σ) bond with three adjacent carbon atoms within the plane. Furthermore, weak van der Waals pi (π) bonds connect the carbon planes in the z‐direction. These two bond types were clearly distinguished in the NEXAFS spectra (Figure 1D). NEXAFS is also instrumental in exploring unoccupied electronic states and verifying the presence or absence of specific bonds.^[^ ^22^ ^]^ The spectrum for pristine graphite (black line) showed two predominant peaks at ≈285 and 292 eV, corresponding to the π^*^ and σ^*^ bonds, respectively.^[^ ^23^ ^]^ In this study, the crystallinity of the milled graphite was compromised due to the disruption of chemical bonds from mechanical stress. Post 4‐h milling, the intensity of the σ^*^ bonds appeared relatively lower than that of the π^*^ bonds. This indicated that the weak van der Waals interactions between the carbon planes were compromised before the in‐plane bonds. After 12 h of milling, the peak intensities for the π^*^ bonds decreased owing to the covalent sigma bond breakdown. Furthermore, the peak position of π^*^ bonds slightly shifted upward. This was attributed to alterations in the bond distance for covalent bonding caused by the disruption of sp^2^ bonding among carbon atoms. The introduction of zirconia and oxygen atoms to the carbon–carbon bonds might also account for this peak shift.^[^ ^24^ ^]^ The peak intensity of the σ^*^ bond consistently diminished beyond 4 h of milling, indicating the breakdown of interplane bonds. The new peak at 288.8 eV was attributed to the formation of oxygen functional groups onto milled graphite. In Figure 1F, the in‐plane crystallite size, *L*_*a*_, denoted the crystallite size along the a‐axis. This value was derived from the Raman spectra using the I_D_/I_G_ ratio according to the following equation:^[^ ^20^ ^]^

(3) $$\;L_{a}=\;\left(2.4\times 10^{-10}\right)\lambda_{l}^{4}{\left(\frac{I_{D}}{I_{G}}\right)}^{-1}$$

where λ_*l*_ (nm) is the wavelength of the Raman emissions, while I_D_ and I_G_ are the intensities of the D and G peaks, respectively. The variation in *L_a_* (calculated from the Raman spectra) was consistent with the variation in *L_c_* (calculated from the XRD data). This correlation further corroborated that the primary change in crystallite size occurred during the initial stages of ball milling.

The relative peak intensity ratio (Figure 1G) of the π^*^ and σ^*^ bonds increased with prolonged milling. This suggests a disruption in the interlayer bonding between the graphite layers. Moreover, the chemical bonding and elemental composition of milled graphite were characterized using X‐ray photoelectron spectroscopy (XPS). Full‐range XPS scans of pristine graphite and 4, 12, and 24 h‐MG graphite are presented in Figure S1 (Supporting Information). These results confirm that the milled graphite primarily consisted of carbon and oxygen.

Based on the material characterizations, the mechanical activation process of graphite through ball milling is divided into three steps, defined by the primary reaction occurring during each interval. This classification drew upon XRD, Raman spectroscopy, and NEXAFS spectroscopy data. The steps were as follows: i) Step 1 (0–4 h milling), decrease in graphite particle size; ii) step 2 (4–12 h milling), augmentation of disorder and distortion of the inner crystalline structure; and iii) step 3 (12–24 h milling), structural saturation and minor restoration. Step 1 entailed exerting mechanical force primarily to reduce particle size, as evidenced by XRD and Raman spectroscopy findings. Step 2 involved disruption of the crystalline structure. The diminished surface area in this stage resulted from particle recombination. This recombination manifested as mere physical adhesion of the fragmented particles without restoration of the long‐range crystalline order. The variances in the reactions between steps 1 and 2 were underscored by shifts in the surface area of graphite.

**Figure 2:**
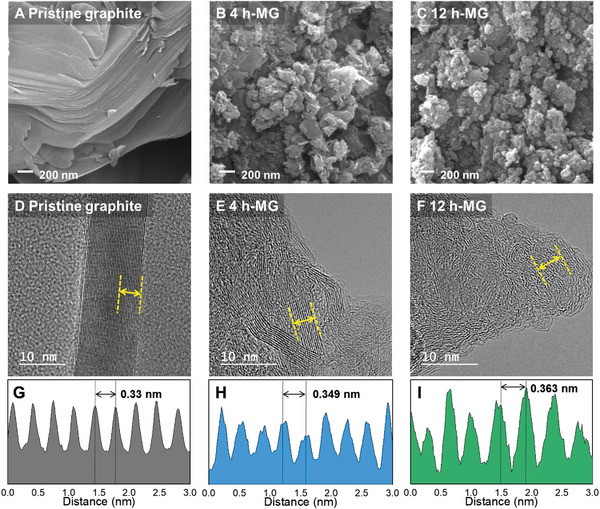
Morphological variations of pristine and ball‐milled graphite. Scanning electron microscopy (SEM) images of A) pristine graphite, B) 4 h‐MG, and C) 12 h‐MG. High‐resolution transmission electron microscopy (HR‐TEM) images of D) pristine graphite, E) 4 h‐MG, and F) 12 h‐MG. G–I) Interlayer spacing of the aforementioned samples.

Morphological and microstructural changes with milling time were investigated using scanning electron microscopy (SEM). The surface morphology of pristine graphite exhibited a typical layered configuration with a smooth surface (**Figure** **2**A). After ball milling for 4 h, the graphite particle grain size became discernible, revealing grains with an average diameter of 100–200 nm (Figure 2B) Furthermore, nanosized flake‐like remnants of the layered structures are observed at the particle edges. The graphite powder underwent a morphological transformation with increasing milling time, while the 12 h‐MG exhibited an agglomerated granular structure with a rough surface (Figure 2C). Figure S2 (Supporting Information) contrasts the morphological features of the 4 and 12 h‐MG. The images confirmed that 4 h‐MG possessed a porous structure, whereas 12 h‐MG comprised aggregated particles.

Transmission electron microscopy (TEM) was employed to further analyze the microstructural properties of graphite at the nanometer scale, as shown in Figure 2D, revealing the highly crystalline layered structure of pristine graphite. After 4 h of milling, the graphite powder was fragmented into small particles, displaying irregular interlayer spacing. This is attributed to the mechanical disruptions that dislodged carbon atoms from their positions during the ball milling process. These displaced atoms repulsively interacted with the remaining carbon atoms, forming a wrinkled morphology (Figure 2E). The 4 h‐MG exhibited disordered regions alongside a local fine‐layered structure. However, the degree of irregularity of the graphite structure became more pronounced with increasing milling time up to 12 h. The local fine‐layered structure vanished in the 12 h‐MG (Figure 2F). Moreover, the 12 h‐MG displayed high internal porosity and highly disordered carbon atoms in its structure. These microstructural alterations influence the interlayer spacing of graphite, which was comprehensively analyzed in this study. Figure 2G shows the characteristic layered structure of graphite, with a consistent interlayer spacing of 0.33 nm. The 4 h‐MG exhibited an irregular structure with an interlayer spacing of 0.35 nm (Figure 2H). Furthermore, the interlayer spacing of the 12 h‐MG (0.36 nm) surpassed that of the 4 h‐MG (Figure 2I). This indicates that the inherent morphology of graphite transformed after 4 h of ball milling, with subsequent alterations in its internal crystalline structure by the 12 h mark. The disintegration of the crystal structure and the evolving interlayer spacings were consistent with the XRD, Raman spectroscopy, and NEXAFS results.

### 2.2 Electrochemical Behavior of Ball‐Milled Graphite

**Figure 3:**
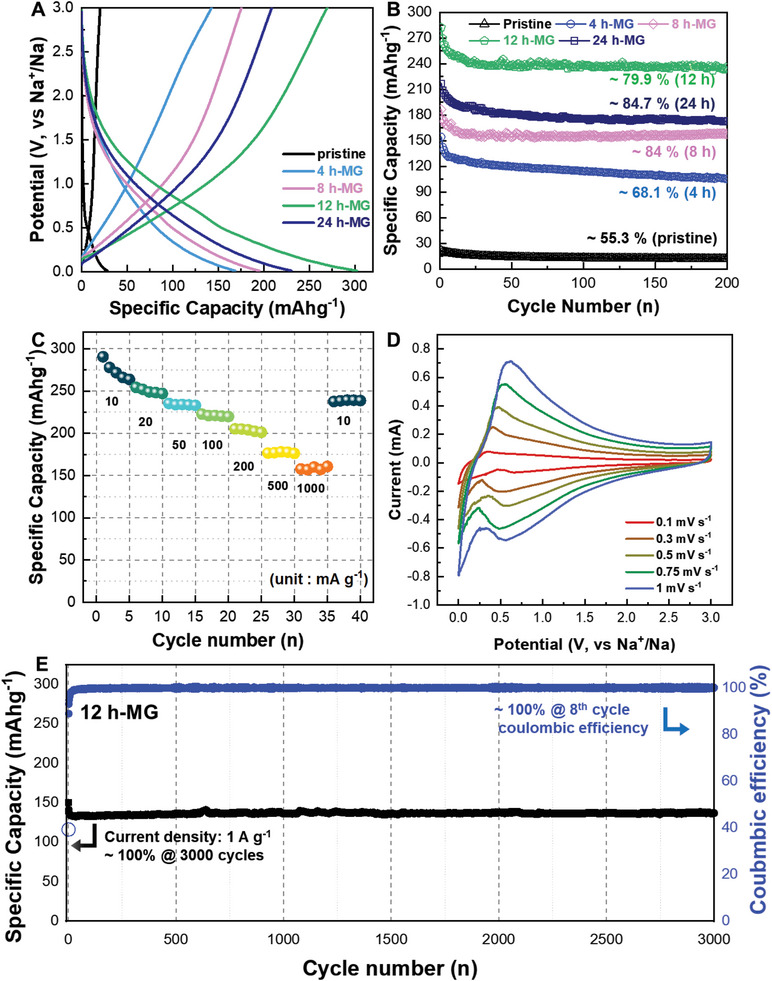
Electrochemical performance analysis. A) Potential profiles of ball‐milled graphite with varying milling times (potential range: 0.01–3 V versus Na/Na^+^, current density: 100 mA·g^−1^). B) Cycle stability of the samples at a current density of 100 mA·g^−1^. C) Rate capability test for the 12 h‐MG over current densities from 10 to 1000 mA·g^−1^. D) Cycling voltammetry (CV) curves of 12 h‐MG at increasing scan rates from 0.1 to 1 mV·s^−1^. E) Long‐term cycling stability test and coulombic efficiency of 12 h‐MG with coulombic efficiency at a high current density of 1 A·g^−1^.

For the electrochemical characterization of the samples, CR2032 coin‐type cells were constructed using 4, 8, 12, and 24 h‐MG, with sodium metal as the working and counter electrodes in a carbonate‐based electrolyte. Notably, various carbonate‐based were used as solvent in SIB electrolytes. Among them, mixture of propylene carbonate (PC) and fluoroethylene carbonate (FEC) solvent exhibited the best cycle retention of 12 h‐MG at a current density of 1 A·g^−1^ (Figure S3, Supporting Information). Accordingly, in our study, NaPF_6_ and PC:FEC were used as the salt and solvent, respectively. **Figure** **3**A displays the voltage profiles of pristine graphite and the 4, 8, 12, and 24 h‐MG during the 2nd galvanostatic charge‐discharge cycle at a current density of 100 mA·g^−1^. The monotonic sloping curves without a plateau region observed across all samples signify varied reaction mechanisms akin to hard carbon. The pristine graphite exhibited a considerably reduced reversible capacity of 21.1 mAh·g^−1^, aligning with prior findings. The 4 h‐MG exhibited an enhanced reversible capacity of 142.7 mAh·g^−1^. The 12 h‐MG delivered the highest reversible capacity of 269.7 mAh·g^−1^ among the ball‐milled graphite samples. Conversely, the reversible capacity for the 24 h‐MG decreased to 208.8 mAh·g^−1^. While the 4 h‐MG possessed the largest BET surface area, the 12 h‐MG exhibited the maximum reversible capacity. Although all milled graphite electrodes displayed similar voltage profiles, their reversible capacities were not proportional to the milling time. Carbonaceous materials with sloping voltage profiles store sodium ions through surface‐dominant reactions, such as insertion/extraction and adsorption. Consequently, the charge/discharge behavior of graphite strongly indicates that the reversible capacity is affected by the surface area and additional factors. Figure 3B illustrates the cycling stability of pristine graphite and the 4, 8, 12, and 24 h‐MG at a current density of 100 mA·g^−1^. Although all samples exhibited decreased reversible capacities over the initial five cycles, they maintained stable cycling performance up to the 200th cycle. Since the 12 h‐MG consistently achieved the highest reversible capacity throughout all cycles, it was selected for further electrochemical evaluations. Figure 3C shows the rate capability test results for the 12 h‐MG, demonstrating reversible capacities of 290.5, 254.3, 235.2, 222.6, 205.2, 176.6, and 157.7 mAh·g^−1^ at current densities of 10, 20, 50, 100, 200, 500, and 1000 mA·g^−1^, respectively. After a discharge/charge cycle at a current density of 1 A·g^−1^, its capacity recovered to 237.6 mAh·g^−1^ when the current density was reduced to 10 mA·g^−1^. These results indicated the remarkable rate capability of the 12 h‐MG, sustaining a capacity retention of 52% despite a 100‐fold increase in current density (from 10 mA·g^−1^ to 1000 mA·g^−1^). Figure 3D presents the cyclic voltammetry (CV) curves for the 12 h‐MG, recorded at escalating scan rates from 0.1 to 1 mV·s^−1^. The CV curves for the 12 h‐MG exhibited broad cathodic and anodic peaks with a minimal voltage offset of ≈0.2 V. Moreover, the negligible peak shift with a 10‐fold increase in the scan rate highlighted the superior electrochemical kinetics of the 12 h‐MG. The long‐term cycling stability of the 12 h‐MG was evaluated at a high current density of 1 A·g^−1^ (Figure 3E). Although a minor drop in capacity was observed at the 30^th^ cycle, the high reversible capacity of 130 mAh·g^−1^ was preserved up to the 3000th cycle despite the high current density (1 A·g^−1^). This indicates the robust cycle stability of the 12 h‐MG. The coulombic efficiencies following the 1st, 2nd, and 8th cycles were ≈40, 90, and 100%, respectively.

### 2.3 Sodium‐Ion Storage Mechanism of Ball‐Milled Graphite

To investigate the sodium‐ion reaction mechanism within graphite, 4 and 12 h‐MG were further analyzed. This was because the 4 h‐MG had a higher BET surface area than the 12 h‐MG, while the 12 h‐MG presented more defects than the 4 h‐MG (Figure S4, Supporting Information). When examining the internal structures of these samples, the d‐spacing of the 4 h‐MG slightly deviated from that of graphite, while the d‐spacing in the 12 h‐MG was larger than that in graphite. Consequently, 4 and 12 h‐MG were compared to determine the factors influencing the sodium‐ion storage reaction mechanism (Figure S5, Supporting Information). Sodium‐ion storage can occur through either a bulk diffusion‐limited reaction or a surface‐oriented capacitive reaction, contingent upon the reaction site. The relative contributions of these mechanisms can be discerned from CV measurements at increasing scan rates according to the following formula:

(4) $$i=a\;\nu^{b}$$

where i denotes the current, ν is the scan rate, and b holds values of 0.5 and 1 for bulk‐diffusion limited reactions and surface‐oriented capacitive reactions, respectively. According to the CV curves of 4 and 12 h‐MG (Figure S5B,D, Supporting Information), the b values for the 12 h‐MG (0.85 and 0.88, Figure S5E, Supporting Information) were greater than those for the 4 h‐MG (0.73 and 0.83, Figure S5C, Supporting Information). This demonstrates a surface‐focused reaction mechanism for sodium ions in the 12 h‐MG. However, the 12 h‐MG had a smaller BET surface area than the 4 h‐MG. Therefore, defects in graphite have a more pronounced impact on surface reactions than the surface area. These results align with previous studies on hard‐carbon‐based SIB anodes, highlighting that surface defects can enhance sodium ion storage, resulting in an increased capacity in the sloping region.

Ex situ XPS depth analysis (Figure S6, Supporting Information) was performed to elucidate the sodium‐ion reaction mechanism within the surface and bulk regions of the samples. The sodiated/desodiated coin cells were placed in a glove box under an inert atmosphere and transferred to an XPS chamber under Ar protection. Each sample underwent Ar^+^ ion beam etching, with XPS repeated at 1 min intervals.

These results align with previous studies on hard‐carbon‐based SIB anodes, highlighting that surface defects can enhance sodium ion storage, resulting in an increased capacity in the sloping region. Moreover, it indicates that oxygen atoms participate in the reversible sodium ion storage reaction in the bulk region. XPS analysis was performed to analyze the reaction.

**Figure 4:**
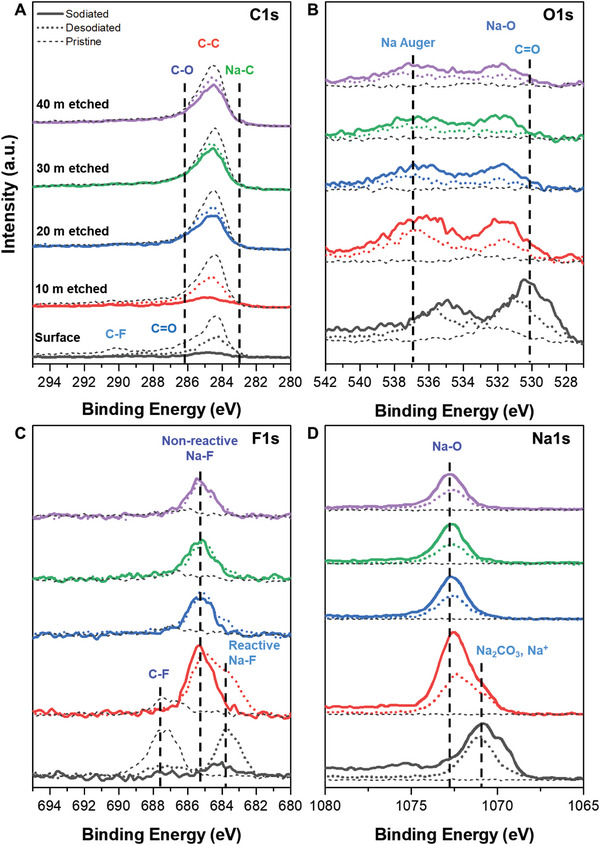
XPS depth profile of 12 h‐MG with Ar^+^ ion etching from surface to 40 mins etched: A) C_1s_, B) O_1s_, C) F_1s_, and D) Na_1s_.

**Figure** **4** shows the ex situ XPS spectra deconvolutions for the 12 h‐MG through a depth analysis. The gray, solid, and dotted lines denote the pristine samples before the electrochemical reaction, the sodiation state (0.01 V vs Na/Na^+^), and the desodiation state (3 V vs Na/Na^+^), respectively. The depth profiles of the C_1s_ spectra for the 12 h‐MG at the surface and after 10, 20, 30, and 40 min of etching are shown in Figure 4A. The surface of the pristine 12 h‐MG exhibited a typical disordered carbon structure with oxygen functional groups. Additionally, three distinct peaks at 284.5, 286, and 290 eV were observed, corresponding to C─C, C─O, and C─F (polyvinylidene fluoride, PVDF), respectively.^[^ ^25^ ^]^ These peaks vanished post‐sodiation owing to the insertion of sodium and oxygen atoms into the carbon matrix. However, these carbon peaks reappeared in the desodiation state. No peaks indicative of direct Na‐C bonds were detected. The O_1s_ spectra remained consistent through the sodiation/desodiation processes, as evidenced in the C_1s_ spectra (Figure 4B). The intensity of the peak at ≈ 532 eV, corresponding to ─O bonding in the bulk region, increased after sodiation.^[^ ^26^ ^]^ However, the carbon‐oxygen peak (286 eV) was not observed in the sodiated state in the C_1s_ spectra. Therefore, the O_1s_ spectra indicated the absence of oxygen bonding. Accordingly, the peaks were inferred to represent sodium‐oxygen (Na─O) bonds, reminiscent of those in Na_2_O. The peak at 530.1 eV was assigned to sodium carbonate (Na_2_CO_3_), which was observed on the surface of the sample.^[^ ^27^ ^]^ The F_1s_ spectrum of the surface for the pristine 12 h‐MG (Figure 4C) shows a distinct peak at 687.6 eV, corresponding to the C‐F bond in PVDF.^[^ ^28^ ^]^ While most peaks disappeared after sodiation, the peak at 683.7 eV reappeared post‐desodiation. This is attributed to the reversible formation of sodium fluoride (NaF), which will be elaborated upon using ex situ NEXAFS spectroscopy. The F_1s_ spectrum of the bulk region exhibited a stable peak at 685.1 eV, confirming the presence of an inorganic trapped sodium compound. The Na_1s_ spectra of the surface of the 12 h‐MG (Figure 4D) were deconvoluted to distinguish the peaks of Na_2_CO_3_ (1071.7 eV) and metallic Na (1071.0 eV).^[^ ^29^ ^]^ These peaks suggested the reversible formation of Na_2_CO_3_ on the surface of the 12 h‐MG during charging. The metallic sodium peak was correlated with sodium adsorption onto the micropores in the low‐potential region. The Na_1s_ spectrum of the bulk region revealed a reversible peak at 1072.6 eV that corresponds to Na_2_O.^[^ ^30^ ^]^ Therefore, the bulk reaction within the 12 h‐MG resulted in the formation of Na‐O compounds without involving sodium ion intercalation. The proposed mechanism was evaluated using ex situ XRD (Figure S8, Supporting Information). No shift was observed in the position of the (002) peak in the sodiated or desodiated states, suggesting that sodium ions did not intercalate between the graphite layers.

Ex situ XPS analysis confirmed the primary reaction between ball‐milled graphite and sodium ions occurs at the surface. However, accurately analyzing these surface reactions with XPS is challenging. As a result, we employed ex situ NEXAFS spectroscopy for detailed surface reaction analysis. NEXAFS provides a robust method for assessing the chemical state of surfaces, and its energy range is aptly suited for examining carbon and oxygen, crucial elements in carbon‐based sodium‐ion battery anodes. The milled graphite samples underwent galvanostatic charging/discharging under a current density of 50 mA·g^−1^, subsequently transferred to the NEXAFS chamber under an argon atmosphere to prevent air exposure. The electrode was fabricated without a carbon‐conducting agent to minimize the detection of unrelated chemical bonds. NEXAFS measurements were performed in the total electron yield (TEY) mode, targeting a detection depth of ≈2 nm. The NEXAFS carbon K‐edge spectra during the initial sodiation phase of pristine graphite, 4 h‐MG, and 12 h‐MG were measured and compared, as shown in Figure S10 (Supporting Information). The powder NEXAFS spectra of the samples before sodiation revealed two predominant peaks corresponding to the σ and π bonds (Figure 1E). The intensities of these peaks gradually diminished during the initial sodiation step, eventually vanishing upon sodiation completion (Figure S10B, Supporting Information). New peaks emerged at ≈288 and 290 eV, superseding the peaks previously corresponding to the σ and π bonds, respectively.^[^ ^23^ ^]^ When the pristine graphite was completely sodiated to 0.01 V, the intensities of σ^*^ and π^*^ peaks negligibly changed, and faint new peaks became discernible. Conversely, the σ^*^ and π^*^ peaks of the 4 h‐MG rapidly vanished upon sodiation, and distinct new peaks appeared (Figure S10C, Supporting Information). A subtle distinction was observed between the carbon K‐edge spectra of 4 and 12 h‐MG. Specifically, a diminutive initial peak of the 4 h‐MG persisted post‐complete sodiation, whereas, for the 12 h‐MG, all original peaks disappeared. This suggested that the 12 h‐MG underwent greater electrochemical activation than the 4 h‐MG. Additionally, the 4 h‐MG showed two sharp peaks of comparable intensities at 0.38 V, while for the 12 h‐MG, the peak intensity at 290 eV was higher than that at 288 eV. The energy range between 288.5 and 290.2 eV in the NEXAFS spectra is still not fully identified.^[^ ^31^ ^]^ The origin of these two peaks is discussed in the subsequent section, drawing comparisons with control samples and previous reports. The peaks at 288.5 and 290.2 eV corresponded to oxalate (deriving from sodium oxalate (Na_2_C_2_O_4_) and carbonate (originating from Na_2_CO_3_), respectively, principal constituents of the SEI layer. When the 12 h‐MG was sodiated to 0.8 V during the first cycle, the oxalate and carbonate peaks were prominent, signifying the SEI layer formation.^[^ ^32^ ^]^

The oxygen K‐edge spectra mirrored the carbon K‐edge spectra during the first cycle (Figure S10D–F, Supporting Information). The 4 and 12 h‐MG showed similar sharp peaks at 533 eV and broad peaks spanning from 539–545 eV. Conversely, the pristine graphite exhibited peaks of minimal intensity. These peaks were assigned to Na_2_C_2_O_4_ (532.9 and 544 eV) and Na_2_CO_3_ (534.6 and 539.8 eV), stemming from the SEI layer formation on the sample surfaces. Furthermore, the oxygen k‐edge spectra of the 4 h‐MG showed sharp peaks at 1.5 V, whereas the 12 h‐MG exhibited a less‐defined peak. This irregularity in the latter was ascribed to an incompletely formed SEI, hinting at the correlation between SEI formation and the sample surface area. Since the 4 h‐MG possessed the highest surface area, it readily formed an SEI layer on its surface. Accordingly, the abundant SEI layer on the 4 h‐MG resulted in the lowest initial efficiency among the samples. However, the fluorine K‐edge spectra exhibited characteristics distinct from the carbon and oxygen K‐edge spectra (Figure S10G–I, Supporting Information). For instance, the fluorine K‐edge spectra displayed the highest and lowest intensities at 0.8 and 0.01 V, respectively. This pattern contrasts with the behaviors observed in the carbon and oxygen spectra.

**Figure 5:**
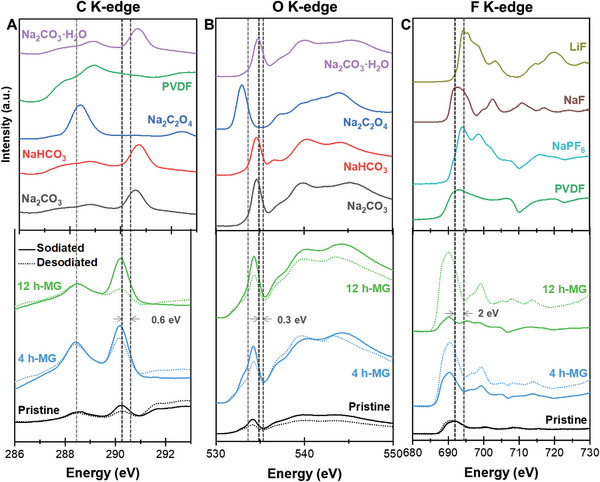
Ex situ NEXAFS measurement at the 2nd sodiation/desodiation state of 12h‐MG with reference materials. A) Carbon K‐edge, B) oxygen K‐edge, and C) fluorine K‐edge. Solid lines represent the fully sodiated condition, while dashed lines correspond to the desodiated state.

To investigate the chemical composition on the surface of graphite, Ex situ NEXAFS were performed in **Figure** **5**. In Figure 5A, the carbon K‐edge spectra of the sodiated and desodiated electrodes (pristine graphite, 4 h‐MG, and 12 h‐MG) are juxtaposed with various carbon‐based reference materials. The sharp peak at 288.5 eV for the sodiated and desodiated electrodes was attributed to oxalate (derived from Na_2_C_2_O_4_), differentiating them from the carbon K‐edge spectra of the reference materials. Furthermore, this stable oxalate peak remained consistent in intensity in the sodiated and desodiated states. The peak at 290.2 eV was assigned to the carbonate bond, displaying varied intensities pre and post‐sodiation across all samples. Therefore, the peak was inferred to contribute to the energy storage mechanism. In this study, the 12 h‐MG exhibited more pronounced peak intensity changes than the 4 h‐MG and pristine graphite, suggesting that defects contribute more to electrochemical activity than the surface area. Moreover, the carbonate peak shifted to 289.6 eV, indicating an elongation in the atomic bond length of the carbonate. Therefore, the bonds might weaken compared to those in pristine Na_2_CO_3_.^[^ ^24^ ^]^ Meanwhile, the peak position for the nonreactive oxalate was identical to that of Na_2_C_2_O_4_. Figure 5B shows the oxygen K‐edge spectra of the samples. The primary peak at 534.3 eV for the 12 h‐MG was assigned to Na_2_CO_3_. The red shift of the peak provides evidence of an increased bond length in the carbonate. The peak at 532.9 eV originated from Na_2_C_2_O_4_, while the broad peaks at 539 and 545 eV were attributed to carbonate and oxalate, respectively. The 12 h‐MG demonstrated distinct peak positions in the sodiation and desodiation states. The peak intensity increased during sodiation and decreased in desodiation, similar to the carbon spectra. Conversely, the oxygen K‐edge spectra of pristine graphite exhibited low‐intensity peaks owing to the subdued reactivity of sodium ions. Figure 5C displays the fluorine K‐edge spectra of the samples. The distinct peak at 690.2 eV was indexed to NaF. However, the NEXAFS findings for fluorine markedly diverged from those for other elements. The intensity at 690.2 eV decreased during sodiation and increased during desodiation. The peak intensity fluctuations for the 12 h‐MG were considerably greater than those for the 4 h‐MG. The ex situ NEXAFS results primarily revealed variations in the spectral peaks based on the state of charge (SoC) of the ball‐milled graphite. These peak shifts signified chemical reactions on the SEI surface, given the measurement depth for the TEY mode in NEXAFS spectroscopy was 2 nm. Therefore, the NEXAFS results confirmed the formation of an SEI layer on ball‐milled graphite comprising three main chemicals: Na_2_CO_3_, Na_2_C_2_O_4_, and NaF. These three components undergo distinct reactions during the charge/discharge cycles. Particularly, Na_2_C_2_O_4_ exhibited a low reactivity akin to that of a conventional SEI layer, whereas NaF and Na_2_CO_3_ yielded divergent outcomes. These results were strongly related to the structural and morphological properties of the samples brought about by the ball‐milling duration and resonated with their electrochemical performance. Therefore, we advocate that sodium‐ion storage is favored on defect‐rich carbon surfaces.

**Figure 6:**
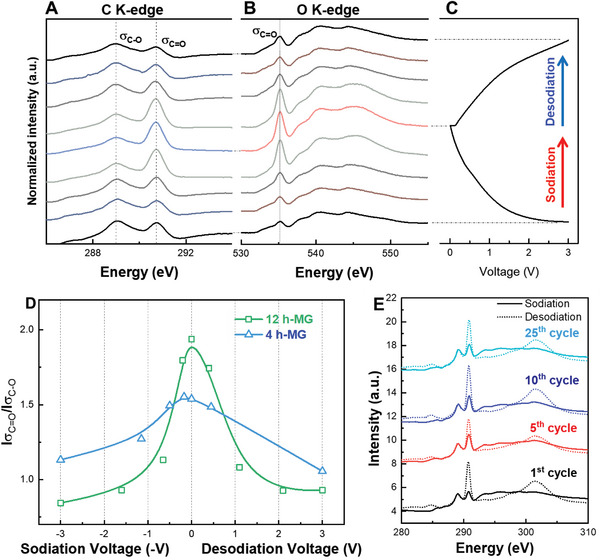
Ex situ NEXAFS measurements of 12 h‐MG in each voltage step during sodiation and desodiation. A) Carbon K‐edge and B) oxygen K‐edge curves. C) Galvanostatic cycling profile at the 2^nd^ sodiation/desodiation cycle. D) Relative intensity between carbonate and oxalate peaks as a function of cell voltage. E) Carbon K‐edge of 12 h‐MG during long‐term cycling.

**Figure** **6** presents the ex situ NEXAFS measurements for the 12 h‐MG, showcasing A) carbon K‐edge and B) oxygen K‐edge spectra at various sodiation/desodiation states during the 2nd cycle. The carbonate peaks of 12 h‐MG demonstrated robust reversibility through repeated charge/discharge cycles. In this study, the voltage range where the carbonate peak was altered was confirmed by determining the relative ratio of the reactive carbonate peak to the stable oxalate peak (Figure 6D). The findings suggested that the carbonate peak changes for the 12 h‐MG were greater than those for the 4 h‐MG in the low‐voltage region. This emphatically pointed to defect‐driven surface reactions within the low‐potential region. Extensive ex situ NEXAFS analysis revealed that the reversible reaction did not originate from an initial side reaction (Figure 6E). Furthermore, the carbonate peak maintained strong reversibility for up to 25 cycles. Additionally, Figure S10 (Supporting Information) shows the NEXAFS spectra of the sodiated and desodiated electrodes, highlighting the surface reactions during discharging and charging.

**Figure 7:**
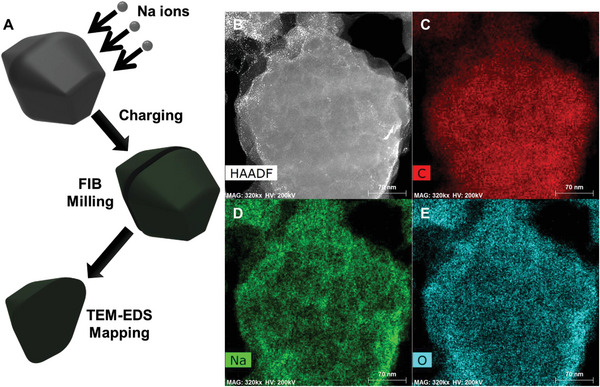
Cross‐sectional TEM analysis. A) Schematic illustration of cross‐section sample preparation. B) Scanning transmission electron microscopy (STEM) image of the cross‐section of 12 h‐MG. Energy‐dispersive X‐ray spectroscopy (EDS) mapping images for C) carbon, D) sodium, and E) oxygen elements.

Ex situ TEM analysis was performed on sodiated 12 h‐MG to further investigate the reaction mechanism of ball‐milled graphite. **Figure** **7**B–E shows the focused ion beam (FIB)‐TEM images using high‐angle annular dark field (HAADF) and energy‐dispersive X‐ray spectroscopy (EDS). Sodium ions were detected on the carbonaceous particle surfaces and within the bulk of the ball‐milled graphite. Therefore, the 12 h‐MG engaged in surface and bulk reactions with sodium ions. The HR‐TEM image (Figure S11A, Supporting Information) of the sodiated 12 h‐MG showed nanocrystalline lattice fringes assigned to Na_2_CO_3_ and NaF. The presence of active sites on the SEI surface (Na_2_CO_3_ and NaF) was confirmed. Nanocrystalline lattice fringes without disrupted carbon fringes were not observed in the HR‐TEM images of the bare 12 h‐MG (Figure S11B, Supporting Information). Elemental distributions across the carbon surface were assessed using TEM‐EDS (Figure S11C–F, Supporting Information). Carbon, oxygen, sodium, and fluorine ions were uniformly distributed on the surface of the 12 h‐MG. Before the electrochemical reaction, fluorine was absent from the bare electrode surface. However, post‐reaction, the TEM‐EDS image displays a uniform fluorine coating on the surface, confirming the presence of NaF on the carbon surface. The structural changes in 12 h‐MG during sodiation and desodiation were investigated using in situ Raman spectroscopy (Figure S12, Supporting Information). The intensity of the D peak decreased during sodiation, reverting to its original intensity following desodiation.

This result contrasts with the ex situ Raman spectra, which indicate the presence of chemically enhanced EG and hard carbon with a high D/G ratio after sodiation. These analyses demonstrate increased structural randomness through the intercalation or insertion of sodium ions into the carbon layer. However, ex situ XRD results indicated that the sodium ion intercalation reaction did not occur within the ball‐milled graphite layers. In situ Raman spectroscopy also indicated a potential intercalation reaction. Consequently, we contend that the sodium‐ion storage mechanism within the graphite layer diverges from prior findings. This necessitates further investigation into graphite as an anode material in practical settings.

**Figure 8:**
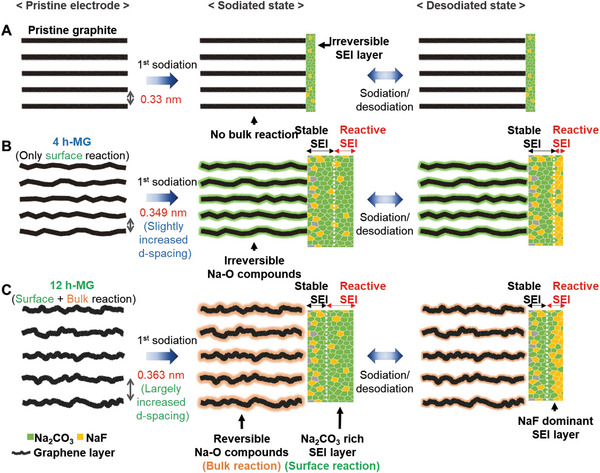
Schematic illustration of the proposed reaction mechanism for A) pristine graphite, B) 4 h‐MG, and C) 12 h‐MG.

We outlined the proposed reaction mechanism for ball‐milled graphite based on the results from various analyses (**Figure** **8**). The sodium‐ion storage reaction between the ball‐milled graphite and sodium ions encompasses surface and bulk reactions. Surface reactions involve sodium ion adsorption onto the carbon, primarily characterizing hard carbon in the sloping‐voltage region. The X‐ray analyses conducted in this research did not identify any adsorption reactions. Furthermore, the NEXAFS measurements verified the occurrence of an unanticipated reversible reaction on the SEI surface of the defect‐rich structure. The ex situ XRD (Figure S8, Supporting Information) and in situ Raman analyses (Figure S12, Supporting Information) indicated that the bulk reaction of ball‐milled graphite was not an intercalation reaction. Instead, sodium ions are introduced into the graphite layer through the formation of Na─O compounds. This bulk reaction is significantly affected by the spacing between graphite layers. When the d‐spacing was insufficient, sodium ions were irreversibly trapped within the graphite layer, lowering the initial efficiency.

Figure 8A illustrates the reaction of pristine graphite with sodium ions, revealing a subdued surface reaction with irreversibly trapped sodium compounds in the graphite layers. While the surface reaction in 4 h‐MG (Figure 8B) was facilitated by adsorption, the bulk region experienced irreversible Na─O compound formation owing to insufficient d‐spacing. Finally, the 12 h‐MG (Figure 8C) exhibited the most dominant surface reaction owing to the reversible formation of Na_2_CO_3_ and NaF. Furthermore, the increased interlayer spacing enabled the reversible formation of Na─O compounds, facilitating the bulk reaction in the 12 h‐MG. However, the detailed mechanism of the bulk reaction remains elusive and warrants further investigation. We anticipate that our findings will provide a foundational perspective on the reaction mechanism of slope‐dominant carbon anodes, lacking a distinct plateau region.

## 3 Conclusion

We explored the mechanical activation of graphite for use as an SIB anode through controlled ball milling. By comprehensively investigating the impact of mechanical energy, variations in the structural and morphological properties we observed and their effects on electrochemical performance relative to milling time were analyzed. The sodium‐ion storage mechanism was investigated using ex situ NEXAFS, XPS, TEM, XRD, and in situ RAMAN. The results from these analyses confirmed the simultaneous occurrence of surface and bulk reactions in the defect‐rich ball‐milled graphite. During sodiation, sodium ions were initially adsorbed on the surface of ball‐milled graphite. Furthermore, the reversible reactions on the SEI surface resulted in alternating formation of Na_2_CO_3_ and NaF owing to the defect‐rich carbon surface. This served as an auxiliary sodium reservoir, enhancing carbon anode performance. In the bulk region, sodium ions integrated through adequate EG layers, forming Na─O compounds. The identified sodium ion storage mechanism significantly diverged from intercalation materials featuring a plateau region, such as hard carbon. This unprecedented storage mechanism provides novel insights for the low‐cost development of slope‐dominant carbon electrodes with defect‐rich structures. This study provides a meticulous examination of the mechanical activation of graphite for high‐performance, cost‐effective sodium ion storage. Furthermore, it introduces a previously uncharted reaction mechanism for defect‐rich carbon electrodes, marked by an unexpected reversible reaction on the SEI surface.

## 4 Experimental Section

### Synthesis of x h‐MG

Mechanically activated graphite was prepared using a straightforward ball‐milling process. A planetary ball‐milling machine (Pulverisette 6, Fritsch) was employed to induce mechanical impact. 10 g of graphite (Sigma–Aldrich) was placed in a zirconia vessel containing 200 g of zirconia balls (9 mm diameter). The vessel was securely sealed under dry air to prevent potential effects from moisture and then agitated at 400 rpm. After the milling, the vessel was cautiously opened in a dry environment.

### Material Characterizations

X‐ray data were collected at the HRPD 9B beamline of the Pohang Accelerator Laboratory (PAL). Raman spectra were measured using a 532 nm laser (Renishaw, England). NEXAFS was conducted at the 10D beamline of PAL to analyze chemical state variations. The surface area was analyzed through nitrogen adsorption‐desorption using the BET method (Autosorb‐iQ 2ST/MP, Quantachrome). The chemical bonding characteristics were evaluated using XPS. Morphological changes in the ball‐milled graphite were observed using FE‐SEM (Inspect F, Thermo Fisher). The microstructural collapse was examined through HR‐TEM (Titan, Thermofisher).

### Electrochemical Characterizations

A composite slurry was prepared from a mixture of ball‐milled graphite, Denka black, and polyvinylidene fluoride (PVDF) in *N*‐methyl‐2‐pyrrolidone (NMP, Alfa Aesar) with an 8:1:1 weight ratio. The slurry was cast onto copper foil (Welcos, Korea) and dried in a vacuum oven at 80 °C. The typical active material loading was 1.2 mg cm^−2^. The coin cell was fabricated using ball‐milled graphite as the working electrode, sodium metal as the counter electrode, a glass fiber separator (Whatman), and a 1 m NaPF_6_ electrolyte in a PC:FEC solvent mixture (98:2 weight ratio). Fabrication was conducted in a glove box under an Ar atmosphere. Galvanostatic charge/discharge tests were performed using a MACCOR series 4000 battery tester after resting for 12 h, within a potential range of 0.01–3.0 V (relative to Na/Na^+^). CV evaluations were conducted using a VMP3 instrument (Bio‐logic Science Instruments) at varying scan rates.

### Ex Situ Characterizations

For ex situ analysis, electrodes were prepared without Denka black (a conducting agent) to minimize side reactions. After cycling, the coin cell was disassembled in an Ar‐filled glove box. The electrode was subsequently rinsed with a pure solvent to remove residual electrolyte. This cleaned electrode was placed on a NEXAFS holder inside the glove box and transferred to a NEXAFS chamber within the 10D beamline at PAL, ensuring no air exposure. Ex situ NEAXAFS was performed in the TEY and fluorescence (FY) modes. For ex situ XPS depth profiling, the cleaned electrode was placed on an XPS holder and transferred to the XPS chamber in a glove box, maintaining an air‐ and moisture‐free environment. Ar^+^ ion sputtering etching and X‐ray measurements were alternated at 1‐min intervals. Cross‐sectional samples for TEM analysis were prepared using FIB milling (Quanta 3D, Thermo Fisher). To prevent contact with air and moisture, a vacuum transfer holder was used for transferring samples between the FIB and TEM systems. TEM‐EDS (energy‐dispersive X‐ray spectrometry) mapping was performed using a Talos TEM (Thermo Fisher Scientific). Selected area diffraction (SAED) patterns were sourced from a JEM‐ARM 200F TEM device. The in situ Raman spectra during the 1st sodiation/desodiation were gathered using a Raman instrument (Renishaw, UK).

## Conflict of Interest

The authors declare no conflict of interest.

## Author Contributions

S.C.L. and Y.H.K. contributed equally to this work. K.Y.C., S.C.L., and Y.H.K. conceived the idea and designed the experiments for the project. S.C.L., Y.H.K., and J.H. prepared the samples and carried out the electrochemical tests. J.‐H.P. and J.‐Y.K. carried out the microscopy analysis and determined physical properties of electrolytes. S.C.L., J.‐H.P., and D.S. conducted synchrotron X‐rays measurement. K.Y.C. and S.C.J. discussed the results. Y.H.K. and J.H. revised the manuscript and supporting information. K.Y.C., S.C.L., and Y.H.K. wrote the paper with contributions from all authors.

## Supporting information

Supporting Information

## Data Availability

The data that support the findings of this study are available from the corresponding author upon reasonable request.
