# Supplementary material for: Mechanical Activation of Graphite for Na‐Ion Battery Anodes: Unexpected Reversible Reaction on Solid Electrolyte Interphase via X‐Ray Analysis
Source: Adv Sci (Weinh). 2024 Apr 26;11(28):2401022. doi: 10.1002/advs.202401022 (PMC11267347; doi:10.1002/advs.202401022)
Supplement: Supplementary file 1 — Supporting Information [file ADVS-11-2401022-s001.pdf]

ADVANCED SCIENCE
Open Access

Supporting Information

for *Adv. Sci.*, DOI 10.1002/advs.202401022

Mechanical Activation of Graphite for Na-Ion Battery Anodes: Unexpected Reversible Reaction on Solid Electrolyte Interphase via X-Ray Analysis

Su Chan Lee, Young Hwan Kim, Jae-Ho Park, Dieky Susanto, Ji-Young Kim, Jonghyun Han, Seong Chan Jun and Kyung Yoon Chung**

Mechanical Activation of Graphite for Na-ion Battery Anodes: Unexpected Reversible Reaction on Solid Electrolyte Interphase via X-ray Analysis

Su Chan Lee†, Young Hwan Kim†, Jae-Ho Park, Dieky Susanto, Ji-Young Kim, Jonghyun Han, Seong Chan Jun and Kyung Yoon Chung**

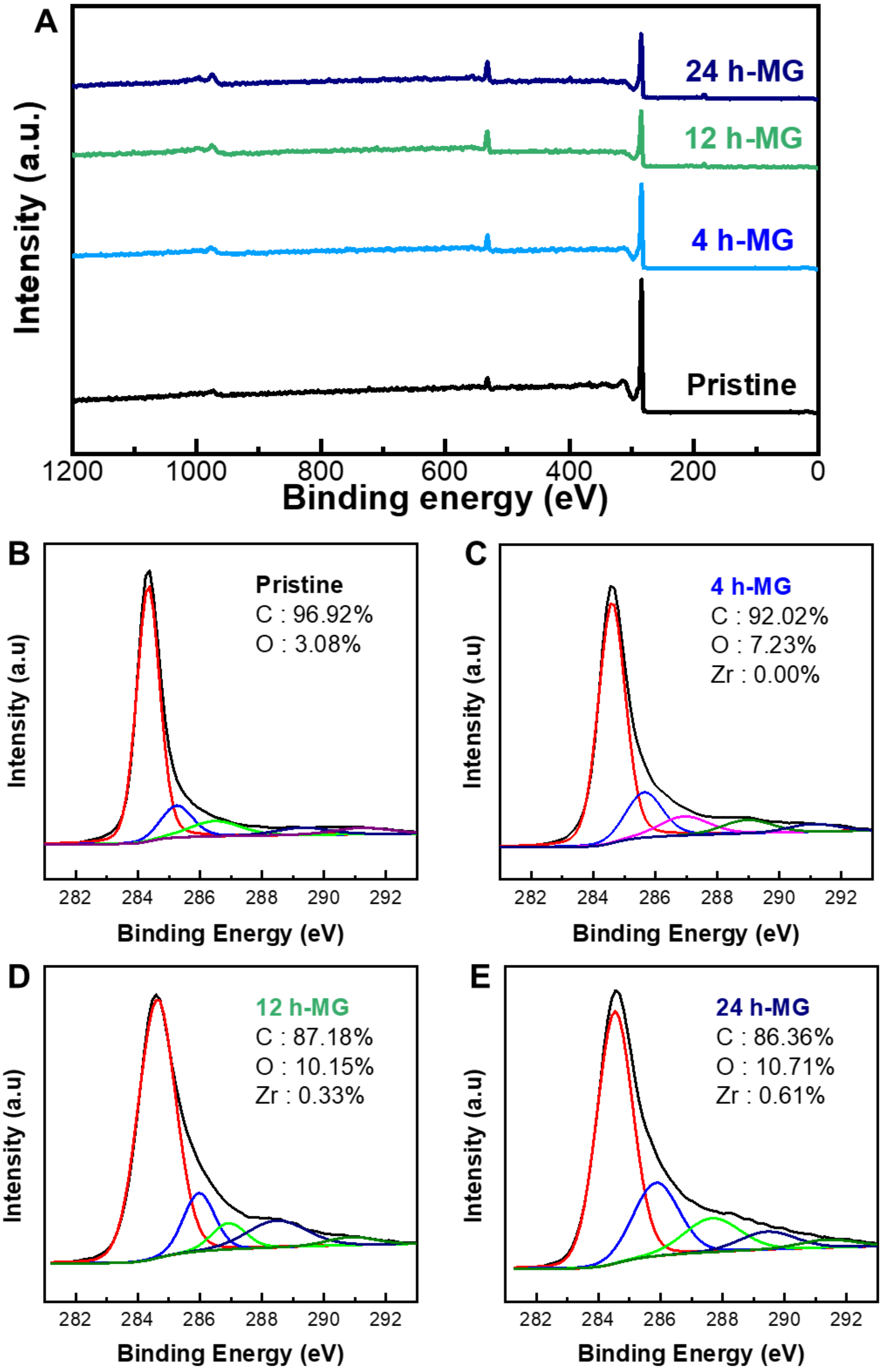

Figure S1. A) Wide-range XPS spectra of pristine graphite, 4 h-MG, 12 h-MG and 24 h-MG. XPS C1S spectra of B) pristine graphite C) 4 h-MG D) 12 h-MG E) 24 h-MG.

Pristine graphite exhibited a distinct C-C bond peak (~284.8 eV) with a minor presence of oxygen functional groups.[1] However, after ball milling, the intensity of the C-C bonds decreased, whereas the number of oxygen functional groups increased. This is consistent with the NEXAFS results. The atomic oxygen concentrations in the samples, calculated from the XPS data, increased from 3.08 % in pristine graphite to 12.15 % in 12 h-MG graphite. As previously highlighted, oxygen originated from the dry air inside the zirconia crucible. As milling proceeded, ZrO_2 partially eroded from the surface of the crucible and balls, leading to a corresponding increase in its concentration. However, after 12 h of milling, the oxygen concentration plateaued due to the exhaustion of available oxygen in the air.

Table S1. Atomic ratios of Pristine, 4, 12 and 24 h-milled graphite.

Sample	C (at. %)	O (at. %)	Zr (at. %)
Pristine	96.92	3.08	N/A
4 h-MG	92.02	7.23	N/A
12 h-MG	87.18	10.15	0.33
24 h-MG	86.36	10.71	0.61

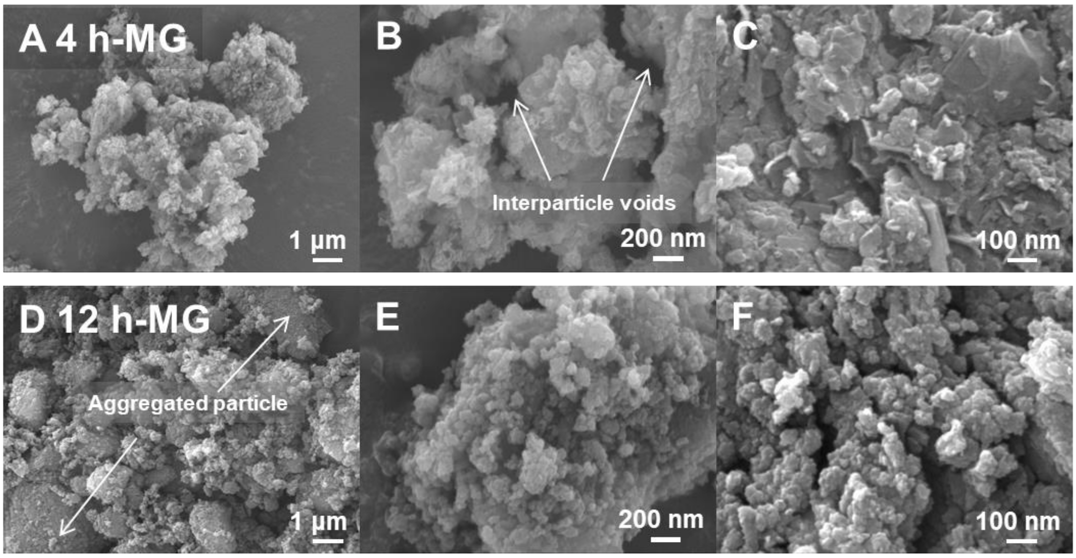

Figure S2. Morphology comparison for the 4 h-MG and 12 h-MG. SEM images of (A-C) 4 h-MG and (D-F) 12 h-MG.

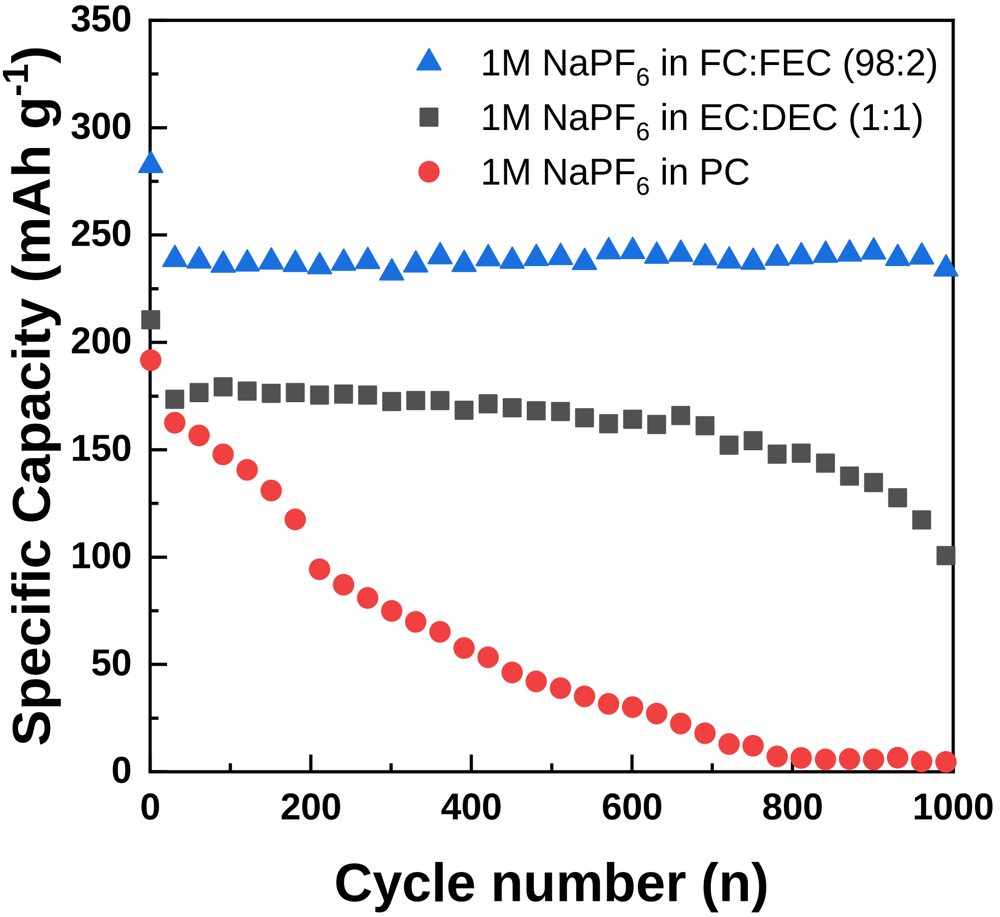

Figure S3. Capacity retention dependent of 12 h-MG on carbonate-based solvents.

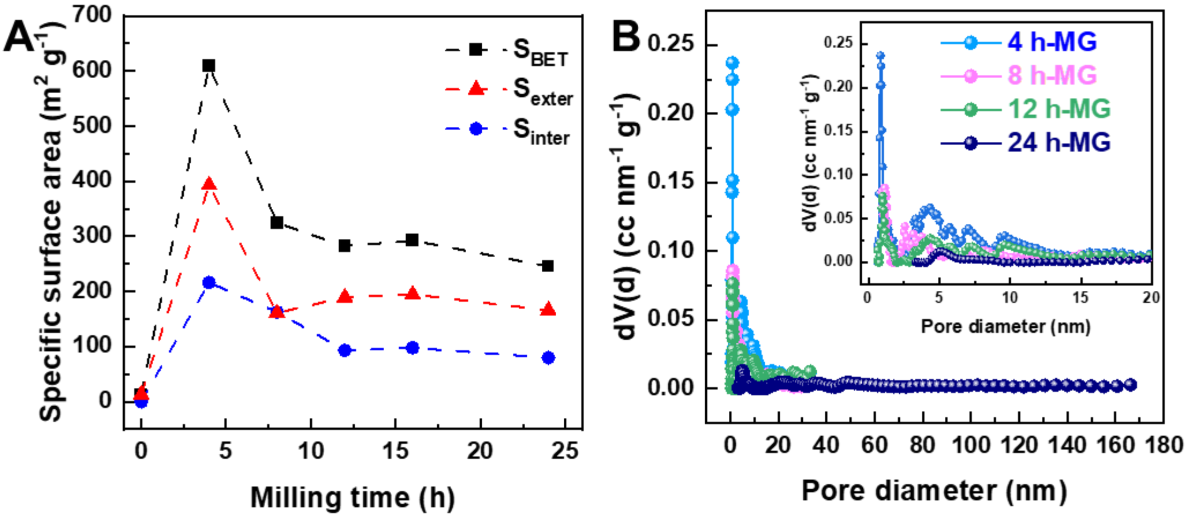

Figure S4 A) N_2 isotherm curves and B) pore size distributions of the samples (inset; highly-magnified pore size distributions)

To assess structural property variations with milling time, N_2 gas adsorption-desorption tests were conducted, characterized by the Brunauer–Emmett–Teller (BET) method **(Figure S4A).** The pristine graphite displayed a BET surface area of 13.2 $m^2 \cdot g^{-1}$. The 4 h-MG presented the highest surface area, reaching 609.0 $m^2 \cdot g^{-1}$. After 4 h of milling, the surface area significantly declined, with minimal further alterations. This suggests that the graphite surface stabilized after 4 h of milling, supporting the findings from XRD, Raman spectroscopy, and NEXAFS analyses. **Figure S4B** displays the pore size distributions for the 4 h-MG, 8 h-MG, 12 h-MG, and 24 h-MG, determined using density functional theory (DFT). The micropore volume for 4 h-MG was the highest compared to the other samples. Furthermore, the micropore volume decreased during steps 2 and 3, due to the limited physical grinding after 4 h of milling, leading to agglomeration of the milled carbon particles.

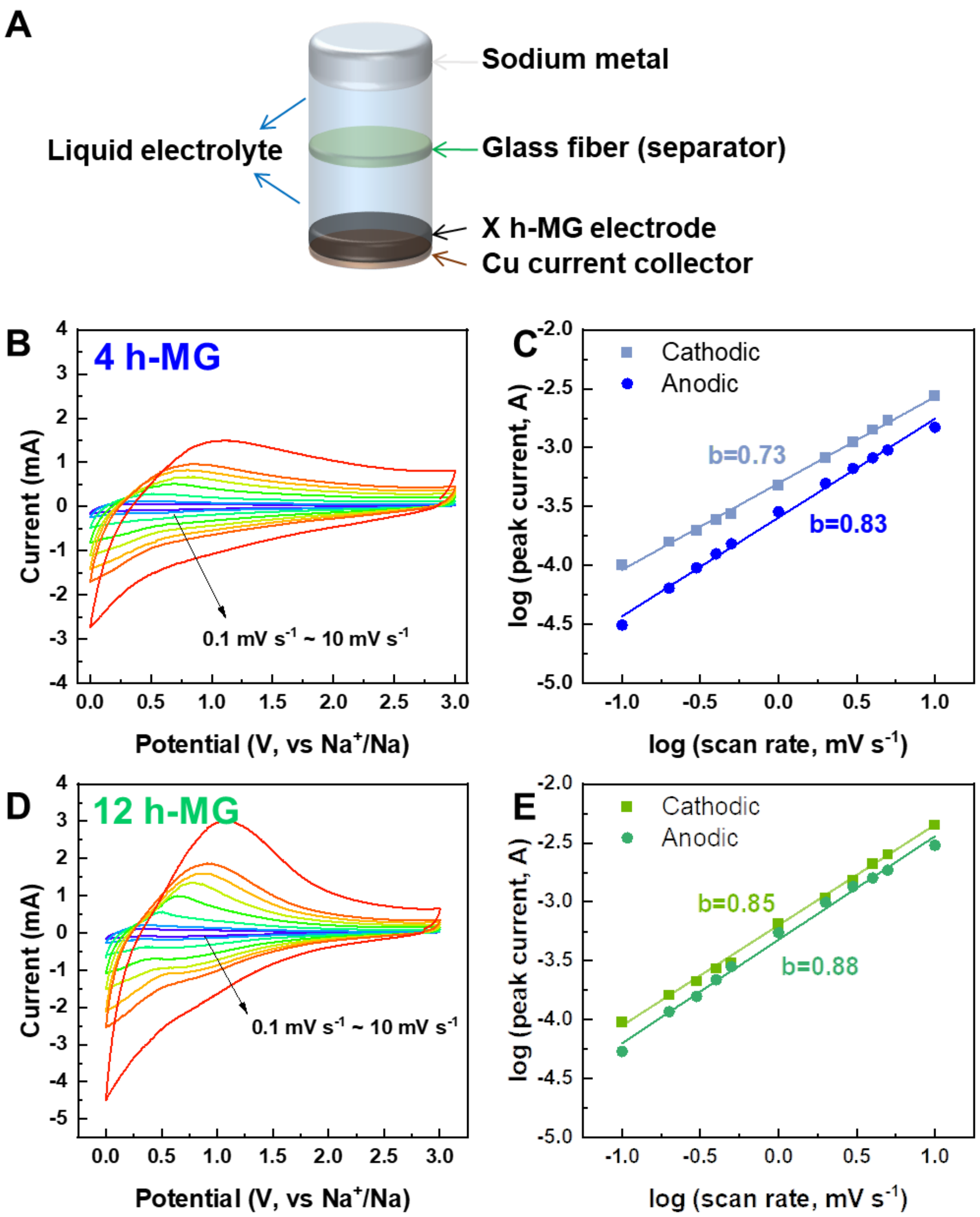

Figure S5. A) Schematic diagram of coin cell. CV curves and b value calculation of (B, C) 4 h-MG and D, E) 12 h-MG.

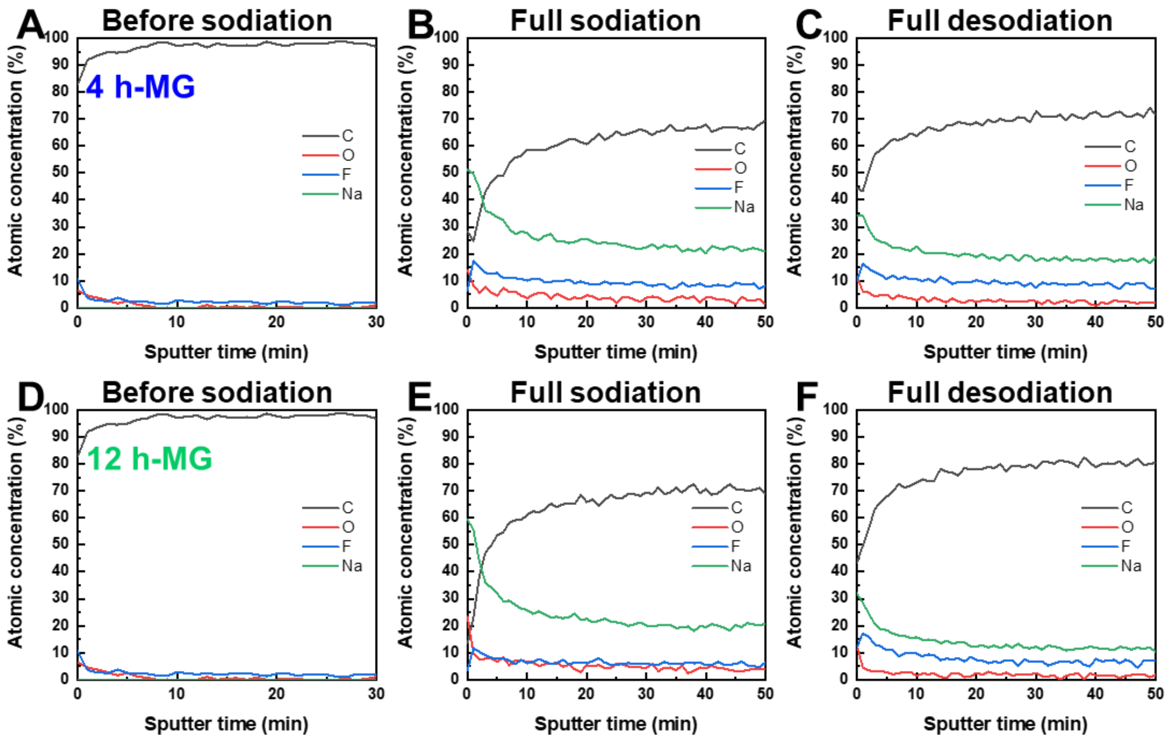

Figure S6. Atomic concentration measured by XPS sputter depth profile before and after sodiation and desodiation states of (A-C) 4 h-MG and (D-F) 12 h-MG.

Figure S6 shows the atomic concentrations of carbon, oxygen, fluorine, and sodium in the 4 h MG, plotted against sputtering time across varying states of charge (SoC). Before the electrochemical reaction, the bare 4 h-MG was primarily composed of carbon at the surface and in the bulk region **(Figure S6A**). After sodiation, **s**odium concentrations at the surface and bulk regions in the inner and outer parts (post 50 min of etching) increased to 51 and 21 %, respectively. This indicates the occurrence of a sodiation reaction throughout the sample (**Figure S6B**). In the desodiated state, Na concentrations were 34.10 % (surface) and 18.92 % (bulk). This was attributed to the irreversible trapping of sodium ions within the structure (**Figure S6C**). The varying Na concentrations between the surface and bulk region during sodiation/desodiation suggest a surface-dominant reaction in the 4 h-MG. Furthermore, according to the atomic concentrations of oxygen and fluorine in the surface and bulk region post sodiation/desodiation, oxygen- and fluorine-containing compounds formed following 4 h of MG sodiation. The change in the atomic ratios of fluorine and sodium indicated that the initial low efficiency of the 4 h-MG originated from forming an SEI at the surface and irreversible reactions in the bulk region. Contrastingly, the 12 h-MG was predominantly carbon-based (**Figure S6D**) before sodiation, akin to the 4 h-MG.

However, the sodium distribution in the sodiated 12 h-MG was 60 % (surface) and 21 % (bulk) **(Figure S6E**), exceeding that in the 4 h-MG. After desodiation (**Figure S6F),** the surface sodium concentration (31 %) of the 12 h-MG was comparable to that of the 4 h-MG. However, the bulk sodium concentration of the 12 h-MG (15 %) was lower than that of the 4 h-MG. This result indicates that the 12 h-MG trapped less irreversible sodium than the 4 h-MG. The variance in sodium storage behavior in the bulk region between the 12 h-MG and 4 h-MG originates from their diverse interlayer spacing and structural properties. Furthermore, despite the extensive surface area of the 12 h-MG, its surface sodium concentration was higher than that of the 4 h-MG, suggesting that sodium storage at the surface was not proportional to the surface area. Moreover, the oxygen concentration in the bulk region of the 12 h-MG significantly increased during sodiation (1.74 %) and desodiation (4.08 %). Conversely, the 4 h-MG displayed consistent oxygen concentrations in its bulk region during the sodiation (1.35 %) and desodiation (2.00 %).

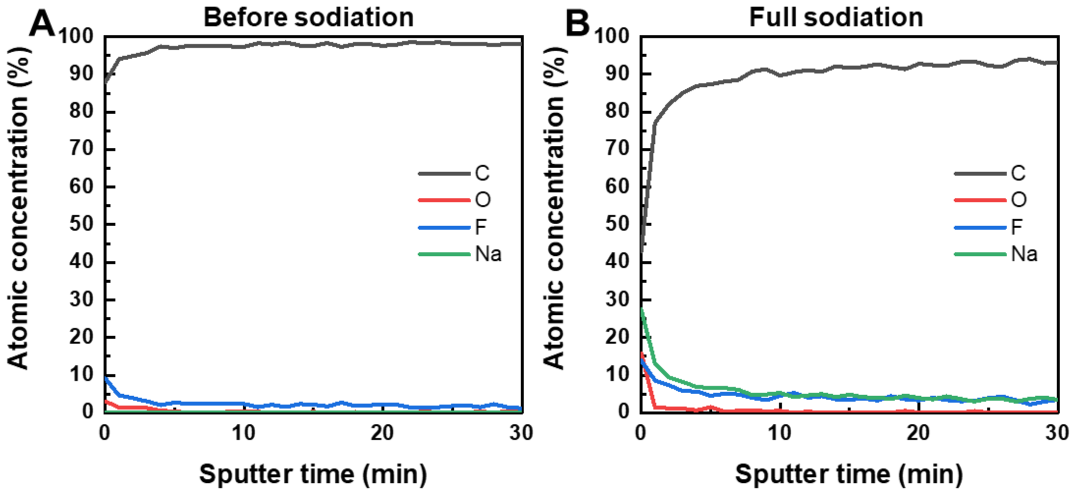

Figure S7. Atomic concentration measured by XPS sputter depth profile A) before and B) after sodiation of pristine graphite.

The depth profile detailing the atomic concentration of pristine graphite was examined before and after sodiation under identical conditions (**Figure S7**). There was no significant increase in Na concentration either on the surface or within the bulk region of graphite, even after sodiation, which is consistent with previous studies.

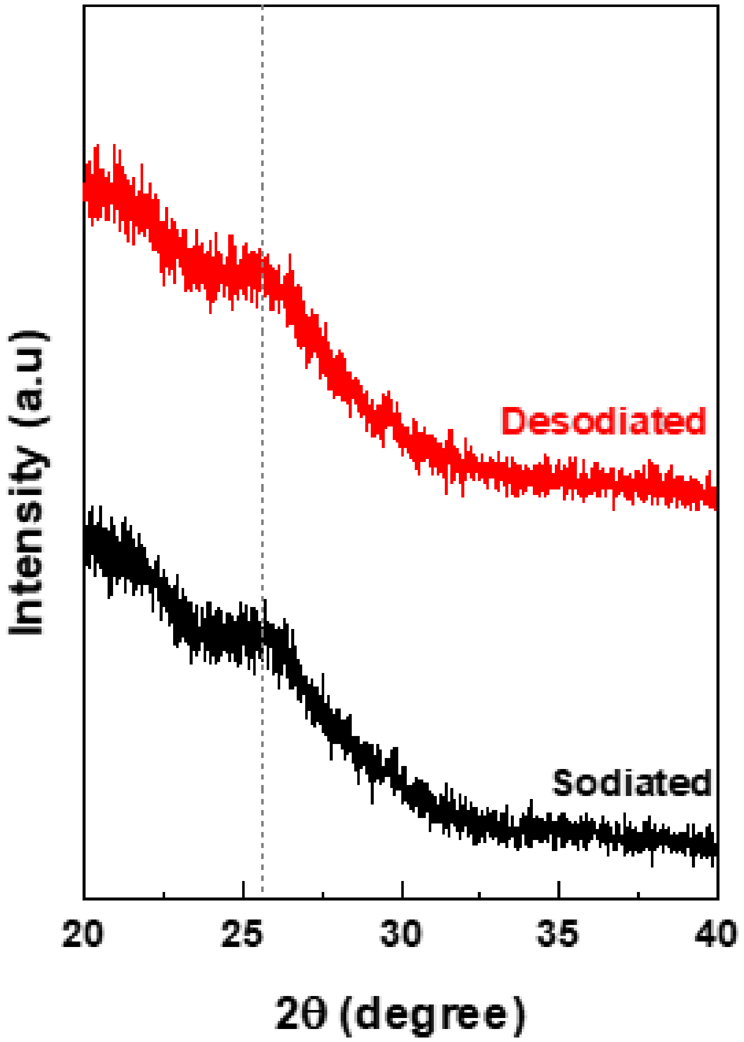

Figure S8. Ex-situ XRD spectra of sodiated and desodiated 12 h-MG.

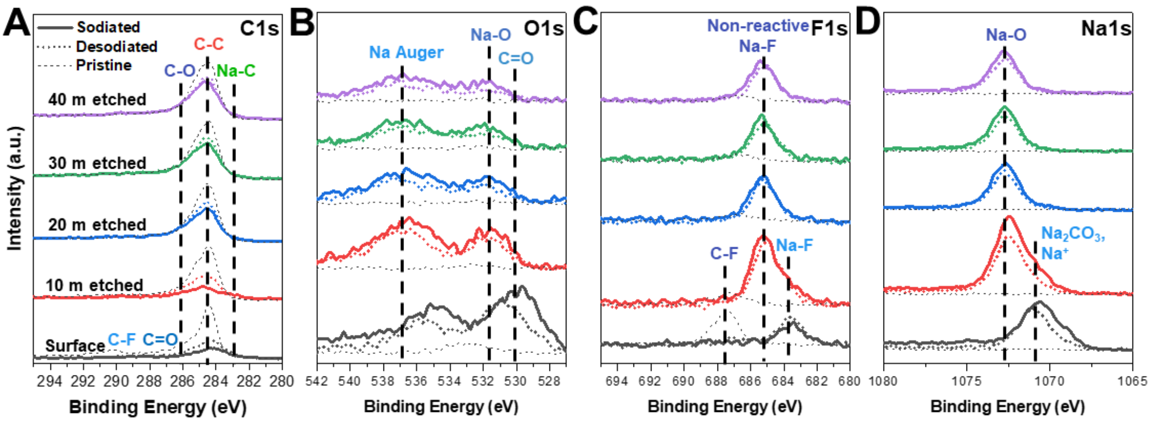

Figure S9. XPS depth profile of 4 h-MG with Ar^+ ion etch from surface to 40 mins etched A) C1s B) O1s C) F1s and D) Na1s

The ex situ XPS profile for 4 h-MG (**Figure S9**) resembled that for 12 h-MG. However, there were significant differences in the C_{1s}, $O_{1s,}$ and Na_{1s} spectra of the sodiated and desodiated states. Therefore, the sodium ion behavior at the surface and bulk regions of the 12 h-MG was more pronounced than that in the 4 h-MG.

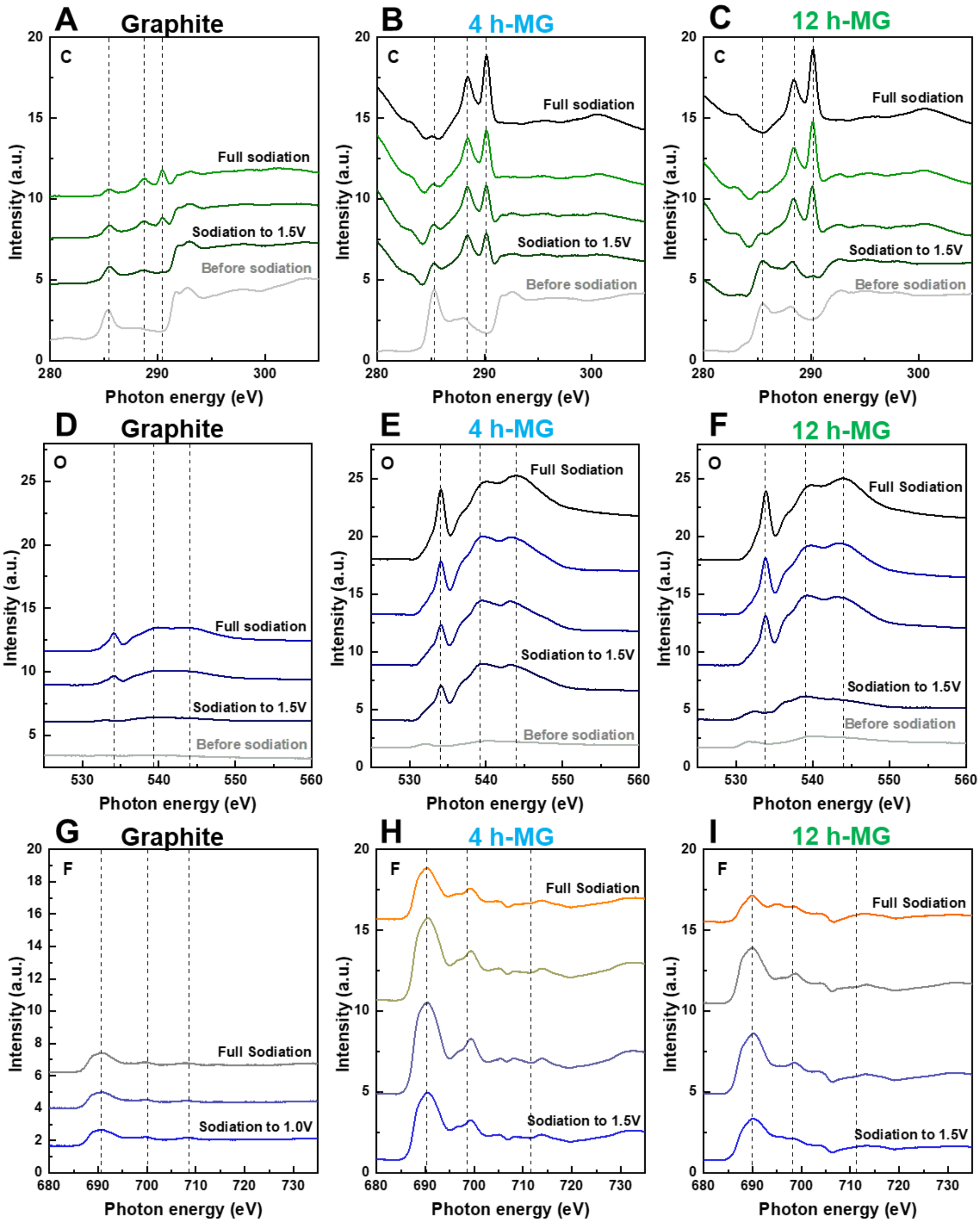

Figure S10. Ex-situ NEXAFS measurement of pristine graphite, 4 h-MG and 12 h-MG. (A-C) Carbon k-edge (D-F) oxygen K-edge (G-I) Fluorine K-edge.

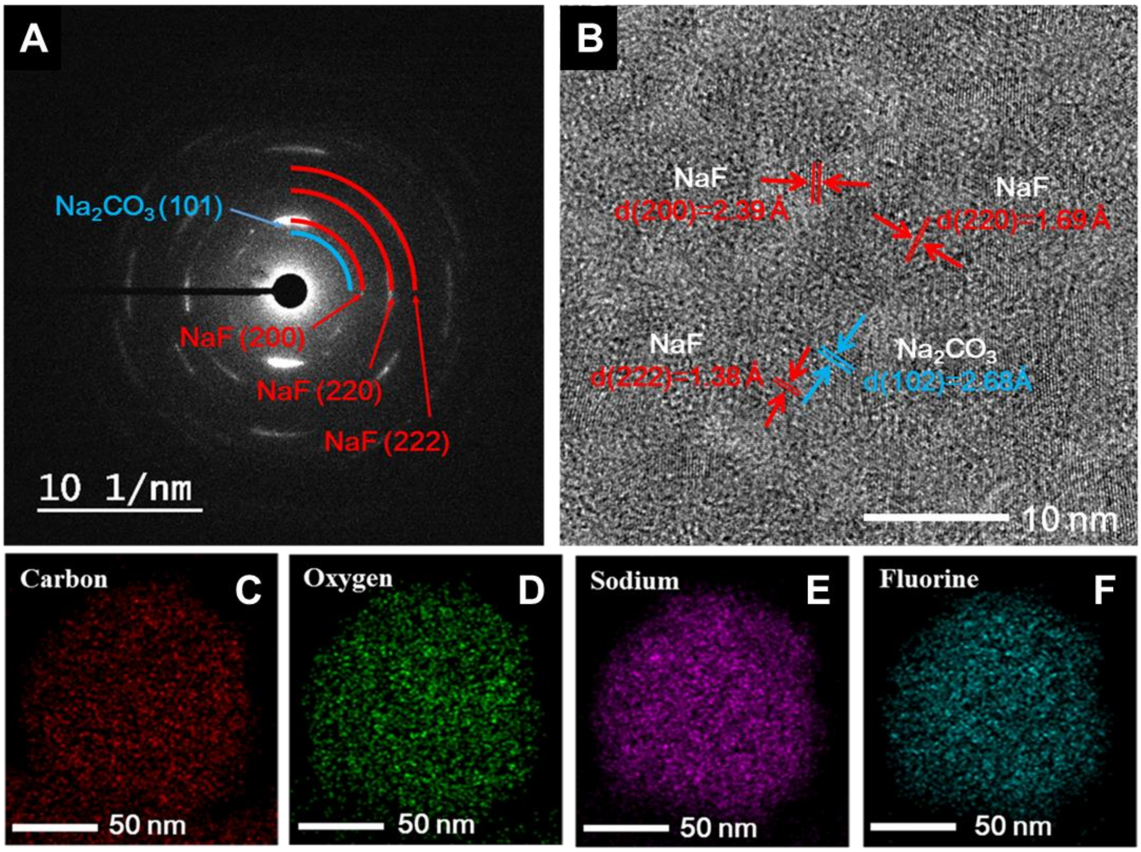

Figure S11. Ex-situ TEM images of fully sodiated 12 h-MG (A-B) HR-TEM images and selected area diffraction (SEAD) patterns. TEM-EDS mapping of C) carbon, D) oxygen, E) fluorine and F) sodium

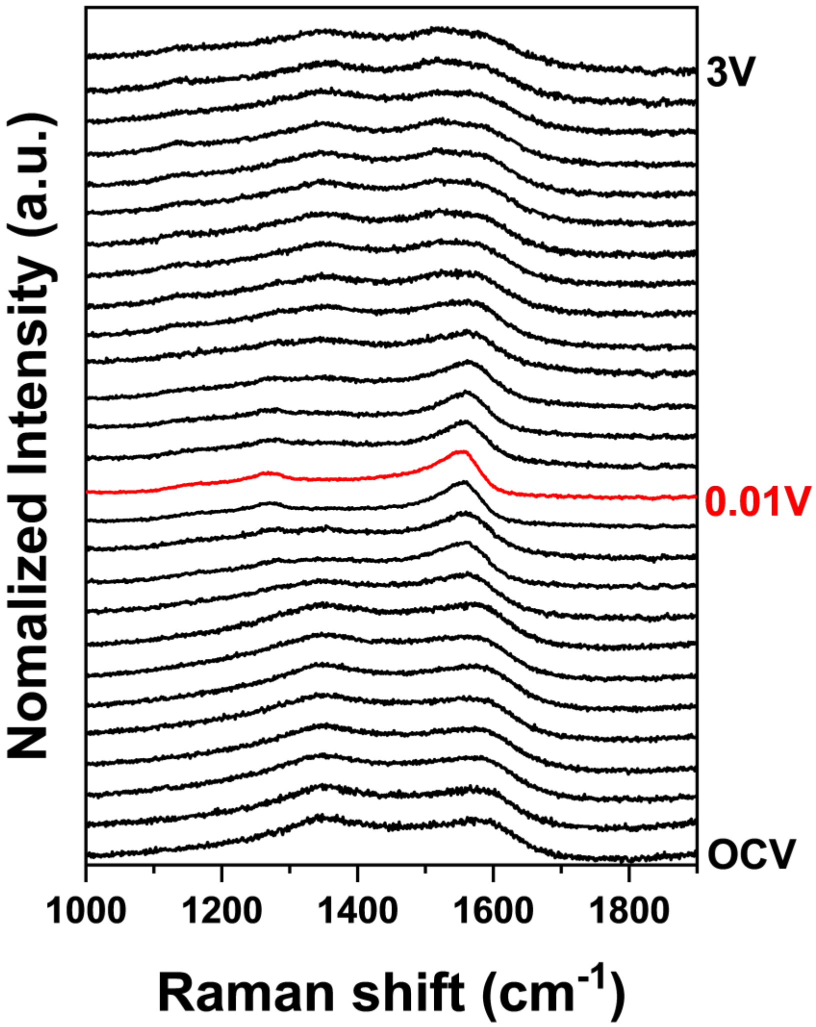

Figure S12. In-situ Raman measurement of 12 h-MG during 1st cycle.

Reference

[1] B. Bouchet-Fabre, E. Marino, G. Lazar, K. Zellama, M. Clin, D. Ballutaud, F. Abel, C. Godet, *Thin Solid Films* **2005**, *482*, 167-171.
